# Supplementary material for: Identifying priority review questions for Cochrane Eyes and Vision: protocol for a priority setting exercise
Source: BMJ Open. 2021 Jun 25;11(6):e046319. doi: 10.1136/bmjopen-2020-046319 (PMC8237719; doi:10.1136/bmjopen-2020-046319)
Supplement: Supplementary data [file bmjopen-2020-046319supp001.pdf]

Appendix 1: Search strategies

Roadmaps and other priority setting exercises (Google)

- roadmap ophthalmology
- unmet need ophthalmology
- priority setting ophthalmology
- prioritization research ophthalmology
- roadmap eye
- unmet need eye
- priority setting eye
- prioritization research eye

Cochrane Central register of controlled trials (CENTRAL)

- exp cataract and exp cataract extraction
- exp amblyopia/
- Strabismus/
- Ocular Motility Disorders/
- exp Nystagmus, Pathologic
- exp diabetic retinopathy
- exp eyelid diseases/
- Lacrimal Apparatus Diseases/
- Lacrimal Duct Obstruction/
- Nasolacrimal Duct/
- exp eye neoplasms
- exp glaucoma
- exp conjunctivitis/
- exp keratitis/
- exp uveitis/
- exp endophthalmitis/
- exp dry eye syndromes
- exp refractive errors
- vision, low
- visually impaired persons"
- blindness
- exp macular degeneration

Evans JR, *et al. BMJ Open* 2021; 11:e046319. doi: 10.1136/bmjopen-2020-046319

Appendix 2: Information for participants / online survey

Cochrane Eyes and Vision Priority Setting Exercise

You are being invited to take part in a research study. Before you decide to take part please read the following information. If you are happy to participate, please tick the box below. Please contact Cochrane Eyes and Vision <email> if there is anything that is not clear or if you would like more information.

Purpose of the study: Cochrane Eyes and Vision plans to undertake a priority setting exercise to assess systematically the nature and extent of research questions relevant to our scope, and to formally incorporate input from a wider range of stakeholders to set priorities for new and updated reviews.

What is involved in taking part in the study? We would like you to take part in two rounds by online questionnaire. On the following pages, you will be presented with a list of potential review topics and asked to rank them in order of importance. These potential review topics were identified by systematic searching of global policy reports, guidelines and reports of relevant reviews and studies. We may have missed important questions and so there will also be an opportunity to tell us of priority topics that are not on the list.

There are separate surveys available for each eye condition (refractive error, cataract, glaucoma, eye problems in children, retinal conditions, outer eye, visual rehabilitation). Please select the condition(s) of interest. This is the first round and we expect it will take around 10 minutes per condition to do so.

Participants who provide their email address will be invited to take part in the second round. In the second round, you will be asked to rank the identified top priority topics according to four criteria. Based on responses to this survey, a final list with the highest ranked top 3 priority topics for each condition will be created.

Confidentiality: All information collected during the course of the research will be kept strictly confidential and will not be shared with anyone outside the research team. This information will be destroyed at the conclusion of the project. Your identity may be known to the researchers if you supply an identifiable email. All responses will be de-identified prior to analysis, and results will be reported at an aggregate level, so that your responses will not be identifiable as belonging to you.

What are the benefits? Participating in this study provides the opportunity to identify priorities for Cochrane Eyes and Vision reviews. These priorities may help guide eye health research and funding in future. Cochrane Eyes and Vision will prioritise the conduct of reviews on topics identified in this process. A summary of this priority setting exercise and the final list of priority topics will be published on Cochrane Eyes and Vision website and through relevant Cochrane channels, as well as in a scientific journal. If you provide your email address, we will also send you this information by email.

What are the risks? There are no risks of physical or psychological harm associated with this process. This first round is anticipated to take 10 minutes of your time at this stage, for each eye condition, and then the subsequent round will take approximately 15 minutes. You will not receive financial or

other type of reimbursement for taking part in the study. Participants who take part in both rounds will be acknowledged as part of group authorship, if they wish.

There is no obligation to take part, and if you do agree to take part you are still free to withdraw at any time and without giving a reason. This study has gained ethical approval from the London School of Hygiene and Tropical Medicine Research Ethics Committee.

Consent: I have read the information above and by ticking this box I consent to be involved in this study. I understand that at any time I may withdraw from this study without give a reason.
