# Supplementary material for: Identifying priority review questions for Cochrane Eyes and Vision: protocol for a priority setting exercise
Source: BMJ Open. 2021 Jun 25;11(6):e046319. doi: 10.1136/bmjopen-2020-046319 (PMC8237719; doi:10.1136/bmjopen-2020-046319)
Supplement: Author's manuscript [file bmjopen-2020-046319.draft_revisions.pdf]

BMJ Open is committed to open peer review. As part of this commitment we make the peer review history of every article we publish publicly available.

When an article is published we post the peer reviewers' comments and the authors' responses online. We also post the versions of the paper that were used during peer review. These are the versions that the peer review comments apply to.

The versions of the paper that follow are the versions that were submitted during the peer review process. They are not the versions of record or the final published versions. They should not be cited or distributed as the published version of this manuscript.

BMJ Open is an open access journal and the full, final, typeset and author-corrected version of record of the manuscript is available on our site with no access controls, subscription charges or pay-per-view fees (http://bmjopen.bmj.com).

If you have any questions on BMJ Open's open peer review process please email info.bmjopen@bmj.com

BMJ Open

Identifying priority review questions for Cochrane Eyes and Vision: protocol for a Priority Setting Exercise

Journal:	*BMJ Open*
Manuscript ID	bmjopen-2020-046319
Article Type:	Protocol
Date Submitted by the Author:	02-Nov-2020

Complete List of Authors:	Evans, Jennifer; London School of Hygiene and Tropical Medicine International Centre for Eye Health<br>Gordon, Iris; London School of Hygiene & Tropical Medicine, International Centre for Eye Health<br>Lawrenson, John; City University of London Division of Optometry and Visual Science<br>Parker, Roses; Oxford University Hospitals NHS Trust, Cochrane MOSS Network<br>Rowe, Fiona; University of Liverpool Department of Health Services Research, Health Services Research<br>Virgili, Gianni; University of Florence Department of Surgery and Translational Medicine; Queen's University Belfast, Department of Public Health<br>Li, Tianjing; University of Colorado, Anschutz Medical Campus<br>Ramke, Jacqueline; London School of Hygiene & Tropical Medicine, International Centre for Eye Health; The University of Auckland, School of Optometry and Vision Science

Keywords:	Medical ophthalmology < OPHTHALMOLOGY, Ophthalmology < SURGERY, Glaucoma < OPHTHALMOLOGY, Cataract and refractive surgery < OPHTHALMOLOGY

SCHOLARONE™ Manuscripts

I, the Submitting Author has the right to grant and does grant on behalf of all authors of the Work (as defined in the below author licence), an exclusive licence and/or a non-exclusive licence for contributions from authors who are: i) UK Crown employees; ii) where BMJ has agreed a CC-BY licence shall apply, and/or iii) in accordance with the terms applicable for US Federal Government officers or employees acting as part of their official duties; on a worldwide, perpetual, irrevocable, royalty-free basis to BMJ Publishing Group Ltd ("BMJ") its licensees and where the relevant Journal is co-owned by BMJ to the co-owners of the Journal, to publish the Work in this journal and any other BMJ products and to exploit all rights, as set out in our licence.

The Submitting Author accepts and understands that any supply made under these terms is made by BMJ to the Submitting Author unless you are acting as an employee on behalf of your employer or a postgraduate student of an affiliated institution which is paying any applicable article publishing charge ("APC") for Open Access articles. Where the Submitting Author wishes to make the Work available on an Open Access basis (and intends to pay the relevant APC), the terms of reuse of such Open Access shall be governed by a Creative Commons licence – details of these licences and which Creative Commons licence will apply to this Work are set out in our licence referred to above.

Other than as permitted in any relevant BMJ Author's Self Archiving Policies, I confirm this Work has not been accepted for publication elsewhere, is not being considered for publication elsewhere and does not duplicate material already published. I confirm all authors consent to publication of this Work and authorise the granting of this licence.

For peer review only

Identifying priority review questions for Cochrane Eyes and Vision: protocol for a Priority Setting Exercise

Jennifer R Evans[1], Iris Gordon[1], John Lawrenson[2] , Roses Parker[3], Fiona J Rowe[4], Gianni Virgili[5,,6], Tianjing Li[7] , Jacqueline Ramke[1,8] and the Cochrane Eyes and Vision Priority Setting Steering Group.

1 International Centre for Eye Health, London School of Hygiene & Tropical Medicine, London, UK

2 City University, London, UK

3 Cochrane MOSS Network, c/o Oxford University Hospitals NHS Foundation Trust, UK

4 University of Liverpool, UK

5 Queen's University, Belfast, UK

6 University of Florence, Italy

7 University of Colorado Anschutz Medical Campus, US

8 School of Optometry and Vision Science, University of Auckland, New Zealand, New Zealand

Corresponding author: Jennifer Evans. International Centre for Eye Health, Department of Clinical Research, London School of Hygiene & Tropical Medicine, Keppel Street, London WC1E 7HT. Jennifer.evans@lshtm.ac.uk 07789674043

Keywords

Eye, Vision, Priority Setting Exercise, Systematic Review, Cochrane

For peer review only - http://bmjopen.bmj.com/site/about/guidelines.xhtml

Abstract

Introduction

Cochrane Eyes and Vision (CEV) is an international network of individuals working to prepare, maintain and promote access to systematic reviews of interventions to treat, prevent or diagnose eye diseases or vision impairment. CEV plans to undertake a priority setting exercise to identify systematically research questions relevant to our scope, and to formally incorporate input from a wide range of stakeholders to set priorities for new and updated reviews.

Methods and analysis

The scope of CEV is broad and our reviews include conditions that are common and have a high global disease burden, for example cataract and dry eye disease, and conditions that are rare but have a high impact on quality of life and high individual cost such as eye cancer. We plan to focus on conditions prioritised by WHO during the development of the Package of Eye Care Interventions. These conditions were selected based on a combination of data on disease magnitude, health care use, and expert opinion. We will collect review questions systematically by (1) contact with key stakeholders, (2) scrutiny of global policy reports, guidelines and other priority setting exercises, (3) ranking of current CEV reviews according to impact, and (4) identifying questions addressed in intervention studies (published and ongoing) in *The Cochrane Library*. For each condition, we will prepare a list of 10-15 potential review questions (new and/or updates). We will seek the views of external and internal stakeholders on this list by conducting an online survey. Equity will be a specific consideration.

Ethics and dissemination

The study has been approved by the ethics committee of the London School of Hygiene & Tropical Medicine (LSHTM). We will disseminate the findings through Cochrane channels and prepare a summary of the work for publication in a peer-reviewed journal.

Article summary

Strengths and limitations of this study:

- This is a systematic assessment of priority questions for Cochrane Eyes and Vision reviews.
- We will seek global input, considering questions irrespective of location or setting.
- The study will draw upon a wide range of stakeholders and resources.
- The focus will be mainly on new intervention reviews and topics.
- The conduct of an online survey will limit the amount of discussion possible.

Introduction

Cochrane Eyes and Vision (CEV) is an international network of individuals working to prepare, maintain and promote access to systematic reviews of interventions to treat or prevent eye diseases or vision impairment. We also conduct systematic reviews of the accuracy of diagnostic tests for common ocular diseases or conditions.

For peer review only - http://bmjopen.bmj.com/site/about/guidelines.xhtml

CEV was established in 1997[1]. We have used a variety of priority setting approaches over the years, including working with guideline panels[2, 3] and participating in a James Lind Alliance Priority Setting Partnership for Sight Loss and Vision[4], but we have largely relied on review author teams to suggest review titles. These titles are evaluated by the editorial base and our network of editors (see https://eyes.cochrane.org/about-cev for ll list) to assess whether they would form a suitable review question , ensuring no overlap with current Cochrane Reviews or with high quality, recently published, non-Cochrane systematic reviews. We consider the following criteria when prioritising review titles suggested by review author teams:

- *Does the proposed new review (or review update) address an important clinical uncertainty?* By "important" we mean that the review topic is one that patients, clinicians, policymakers or the public would like to have answered i.e. is important to them. A "clinical uncertainty" reflects the situation where there is evidence of variation in practice or differing opinions as to the best or most effective intervention.
- *Will a Cochrane Review (new review or review update) at this point in time resolve this clinical uncertainty?* Largely this means that we aim to prioritise reviews that will include a number of reasonably large and robust studies. However, sometimes we judge that identifying a gap in the evidence is also important, if identifying that gap will be likely to lead to further intervention studies.
- *To what extent would resolving this clinical uncertainty reduce the magnitude of vision impairment and eye health disorders?* This is clearly a subjective judgement but takes into account knowledge of the magnitude of the eye disease or vision impairment and anticipated effect, or cost-effectiveness, of the intervention.
- *To what extent would resolving this clinical uncertainty reduce inequalities/disparities in (i.e. have an equalizing effect of) the magnitude of disease or access to care for vision impairment or eye health disorders?* Ideally, we would prioritise reviews and interventions that address inequity.

This approach identifies titles that are important to clinicians and researchers but may be less likely to identify questions relevant to other stakeholders such as patients, public and policy makers. It is also likely to result in focus on higher-income settings, with high research capacity, and less emphasis on equity-relevant titles. A recent review highlighted the lack of equity-relevant Cochrane Reviews on cataract.[5]

CEV plans to undertake a priority setting exercise to assess systematically the nature and extent of research questions relevant to our scope, and to formally incorporate input from a wider range of stakeholders to set priorities for new and updated reviews. This document outlines the protocol for a priority setting exercise to identify important review questions. It is informed by guidance prepared by Cochrane on setting review priorities[6] and by the REporting guideline for PRIority SEtting of health research (REPRISE) framework for reporting priority setting of health research [7]. Equity will be a specific consideration in the priority setting process. As part of this process, we will also be informed by the work of other organisations undertaking priority setting activities in the field of Eyes and Vision, and with whom we are working.

- The International Centre for Eye Health is conducting a Delphi exercise to identify the top grand challenges in global eye health.[8] As part of this exercise, a wide range of

For peer review only - http://bmjopen.bmj.com/site/about/guidelines.xhtml

participants, including practitioners and patients from all regions of the world, have identified the top 40 challenges for Global Eye Health. A grand challenge was defined as *"a specific barrier that, if removed, would help to solve an important health problem. If successfully implemented, the intervention(s) to address this grand challenge would have a high likelihood of feasibility for scaling up and impact*". As part of this process, the Scientific Advisory Board for the Grand Challenges in Global Eye Health will be developing a list of relevant research questions relating to the identified challenges.

- WHO Vision Programme is preparing a package of eye health care interventions. The aim of this package is *"to support Ministries of Health in planning, budgeting, and integrating eye care interventions into health systems."* CEV is collaborating with the WHO Vision Programme to identify high-quality evidence to support these recommendations, which will be prepared by development groups including practitioners from all parts of the world. [9] As part of this process, we will identify gaps where high-quality systematic reviews have not yet been conducted.

Aims and Objectives

CEV Priority Setting Exercise has the following aims:

1. to generate a list of priority topics, for both new and updated reviews;
2. to ensure that our main stakeholders are involved in setting priorities;
3. to publicise the list of review topics generated.

Methods

Context and scope

The scope of CEV is broad and includes conditions that are common and have a high global disease burden, for example cataract and dry eye disease, and conditions that are rare but have a high impact on quality of life and high individual cost such as eye cancer. The scope of CEV is global and is not restricted to any particular geographic area. The intended beneficiaries of our work are people making health-care decisions for eye health care. CEV reviews primarily address questions relevant to clinical research (treatment, diagnosis. prognosis) but also potentially cover public health, health services and implementation research.

We plan to focus on the list of conditions set out in *Box 1*. This list was adapted from the list of conditions prioritised by WHO as part of the development of the Package of Eye Care Interventions (PECI).[9] These conditions have been selected based on a combination of data on disease magnitude, health care use, and expert opinion.

For peer review only - http://bmjopen.bmj.com/site/about/guidelines.xhtml

Box 1: List of conditions

1. Cataract
2. Refractive error
3. Diabetic retinopathy
4. Glaucoma
5. Macular degeneration
6. Amblyopia
7. Disorders of eye movement
8. Infectious and inflammatory diseases
9. Ocular trauma
10. Ocular surface disorders
11. Disorders of the eyelid and lacrimal system
12. Eye cancer

Governance

We have set up a Steering Group to advise on the scope of the exercise including ophthalmologists, optometrists, orthoptists, ophthalmic nurses and relevant professional bodies, patient organisations, experienced clinical editors and information specialists of CEV; it includes participants from high and low-income settings. Members of the team have been involved in previous priority setting exercises.[5, 8]

Stakeholders and participants

Any person with an interest in health-care decision-making relevant to eye health care is eligible to take part. This includes patients, caregivers, the general public, health professions, researchers, policy makers, government and non-government organisations and industry. We will actively seek out potential stakeholders using the expertise of the Steering Group to identify relevant stakeholder organisations and individuals globally in their field. We will then write to each stakeholder organisation or individual identified by the Steering Group to invite them to take part in the online survey and to identify further relevant stakeholders (snowballing).

Identifying research questions

For each of these conditions we will undertake the following steps in order to identify priority questions for each condition, consulting with the Steering Group as needed:

(1) Identify key stakeholders, initially by consultation with the Steering Group, but also including further "snowballing" I.e. asking stakeholders to identify other relevant stakeholders. We will request information on key research needs. The categories of stakeholders that we will consider include but will not be restricted to:

- Patients and public
- Providers, purchasers and payers
- Policy makers
- Principal investigators and research funders

(2) Identify and summarise relevant data on research in Eyes and Vision. We plan to use the following sources:

For peer review only - http://bmjopen.bmj.com/site/about/guidelines.xhtml

- Global policy reports
- Other research prioritisation and roadmaps
- Guidelines
- CEV systematic reviews
- CEV@US Project Database of Systematic Reviews in Eyes and Vision
- Intervention studies on *The Cochrane Library*

We will identify global policy reports and other research prioritisation exercises from searching academic databases and contact with our Steering Group. We will scrutinise these reports for identified research questions. We will identify high-quality guidelines from the process followed by the WHO during the development of the Package of Eye Care Interventions.[9] We will rank current CEV reviews according to impact using Altmetric and access statistics provided by the publisher. The top 20% of reviews identified by this process will be eligible for the priority setting process. We will search *The Cochrane Library* for studies published within the last three years, or on a trials registers. One author (IG) will scan these reports to identify new potential review titles i.e. PICOs identified in two or more studies, that have not already been addressed in Cochrane Reviews.

(3) Using the information from (2) we will prepare a list of potential review topics, removing duplicates as needed or merging/combining similar questions. This list will be reviewed by the Steering Group after it has been refined by removing questions where:

- High quality current systematic reviews exist and new trials are either unlikely to have been done, or if they have been done, are unlikely to change the conclusions of the review. We will check for Cochrane Reviews and non-Cochrane high quality systematic reviews in the database maintained by CEV@US project.
- Topic is beyond scope because either it does not address a condition of interest (*Box 1*) or it does not address a relevant clinical question.
- Question is unclear or ill-defined or cannot be answered by either an intervention, diagnostic test accuracy, prognostic or scoping review.

Conducting the priority setting exercise

We will seek the views of external and internal stakeholders on the list generated in (3) by conducting an online Delphi survey using Qualtrics software (Qualtrics, Provo, Utah USA available at www.qualtrics.com).

Participants will be identified through two methods: advertising and direct invitation. We will advertise through social media (for example Twitter, Facebook and focussing on Cochrane channels) and within academic and clinical networks (for example Community Eye Health Bulletin, Alumni networks). We will inform the stakeholders identified above and ask members of the Steering Group to circulate within their networks. We will invite some participants directly to take part and will ensure that these invitations are balanced with respect to gender, location (working/living in a high-income or lower-income setting) and profession (clinician, patient, policymaker). We will approach Cochrane contributors and other contacts we consider potentially interested to contribute and will draw upon the previous participants of the Delphi exercise for Grand Challenges for Eye Health (names available in public domain). We will not perform any other formal process for ensuring

For peer review only - http://bmjopen.bmj.com/site/about/guidelines.xhtml

balance, but we will collect limited information on respondents (gender, location, profession) so that we understand who has responded.

We will conduct a two-round process. In the first round, we will present questions separately for each condition and ask the panel to rank in order of priority. There will be space for the participant to add in additional questions that have not been included in the presented list. We will be seeking questions relevant to interventions reviews, diagnostic test accuracy reviews, prognostic reviews or scoping reviews only. In the second round, the top five questions for each condition will be presented, along with any additional new questions identified during the course of the first round. Participants will be asked to score the review questions according to the following criteria (4-point scale):

- Does the proposed new review (or review update) address an important clinical uncertainty?
- Will a Cochrane Review (new review or review update) at this point in time resolve this clinical uncertainty?
- To what extent would resolving this clinical uncertainty reduce the magnitude of vision impairment and eye health disorders?
- To what extent would resolving this clinical uncertainty reduce inequalities/disparities in (i.e. have an equalizing effect of) the magnitude of disease or access to care for vision impairment or eye health disorders?

Following these two rounds, for each condition we will identify the top three questions. Each question will be structured in the PICO format, adapted for other contexts for example diagnosis, prognosis as necessary. As part of this process we will be guided by the quantitative results of these surveys but, to align with our commitment to widen the inclusion and equity-relevance of CEV reviews, we will be careful to ensure that questions relevant to lower income settings are prioritised at this stage.

Equity

We will consider equity as part of this process, drawing upon methods developed by the Campbell and Cochrane Equity Methods group (https://methods.cochrane.org/equity/about-us). One member of the Steering Group has a special interest in equity. Our main approach will be to ensure that we have as wide a participation as possible (see above for details). We are taking the opportunity to draw upon current partnerships with global initiatives to ensure priorities are informed by representation from low- and middle- income countries.[8, 9] We will also prioritise questions that the survey participants have considered would reduce inequalities (last question above).

Patient and public involvement

Patient and public involvement will be through the Steering Group and by contributing to the priority setting exercise.

Ethics and dissemination

The study has been approved by the ethics committee of the London School of Hygiene & Tropical Medicine (LSHTM). We will disseminate the findings through Cochrane channels and prepare a summary of the work for publication in a peer-reviewed journal.

For peer review only - http://bmjopen.bmj.com/site/about/guidelines.xhtml

We will publish, through relevant Cochrane channels, our intention to conduct a priority setting process so that external and internal stakeholders may be involved.

We plan to:

- Document our plans for priority setting including stakeholder engagement and methods and criteria.
- Document the implementation of the priority-setting process on our website (including link to relevant network portal) and in an academic publication.
- Publish a list of priority topics on the CEV website.
- Develop a plan for how the priority topics will be delivered.
- Provide feedback to stakeholders involved, including notification when priority reviews are published.

Currency/timeframe

We plan to complete the Priority Setting Exercise during January to March 2021 and repeat within 3 to 5 years.

Evaluation and feedback

Written feedback will be given to all participants in the process who have supplied an email address, including a plain language account of the process and outcome of the process. All participants will be acknowledged in the final report (with permission).

We will evaluate the priority setting process and outcomes as follows.

Short-term evaluation

1. Did the priority setting process meet Cochrane mandatory criteria?
2. Did the priority setting process meet Cochrane desirable criteria?
3. Was the process complete within the pre-specified time frame?
4. Was the process completed without using excessive CEV staff time?
5. Gather feedback from stakeholders
   a. What did stakeholders like about the process?
   b. What did stakeholders want to improve about the process?
6. Gather feedback from CEV staff
   a. What did CEV staff like about the process?
   b. What did CEV staff want to improve about the process?

Long-term evaluation

1. Were the resultant reviews produced in a timely manner?
2. Were the resultant reviews relevant/important? For example, did they have higher Altmetric/impact score?
3. Were the reviews used in guidelines or other policy documents?

Other considerations:

For peer review only - http://bmjopen.bmj.com/site/about/guidelines.xhtml

- Equity – how have the results improved equity? Have any of the reviews considered most relevant for equity in the process above been undertaken?
- Has there been an increase in authors from low- and middle-income settings?
- Sustainability – have the reviews been used to train up new systematic reviewers?

Conclusion

A systematic and transparent approach to identifying review questions, informed by credible evidence, and reaching out to a broader group of people to assess priorities will help CEV establish which reviews need to be prioritised in the next 3 to 5 years.

For peer review only

For peer review only - http://bmjopen.bmj.com/site/about/guidelines.xhtml

References

1. **Evans J, Li T, Virgili G, Wormald R, Cochrane E, Vision: Cochrane Eyes and Vision: a perspective introducing Cochrane Corner in Eye. *Eye* 2019, 33(6):882-886.**

2. **NICE: Age-related macular degeneration: diagnosis and management In: *NICE Guideline NG82.* National Institute of Health and Care Excellence; 2018.**

3. **NICE: Cataracts in adults: management. In: *NICE Guideline NG77.* National Institute of Health and Care Excellence; 2017.**

4. **Rowe F, Wormald R, Cable R, Acton M, Bonstein K, Bowen M, Bronze C, Bunce C, Conroy D, Cowan K *et al*: The Sight Loss and Vision Priority Setting Partnership (SLV-PSP): overview and results of the research prioritisation survey process. *BMJ open* 2014, 4(7):e004905.**

5. **Evans J, Mwangi N, Burn H, Ramke J: Equity was rarely considered in Cochrane Eyes and Vision systematic reviews and primary studies on cataract. *Journal of clinical epidemiology* 2020, 125:57-63.**

6. **Cochrane: Guidance note for Cochrane Groups to define systematic review priorities. In.; 2019.**

7. **Tong A, Synnot A, Crowe S, Hill S, Matus A, Scholes-Robertson N, Oliver S, Cowan K, Nasser M, Bhaumik S *et al*: Reporting guideline for priority setting of health research (REPRISE). *BMC medical research methodology* 2019, 19(1):243.**

8. **Ramke J, Faal H, Burton M: Take part in the Grand Challenges in Global Eye Health study. *Community eye health* 2019, 32(106):36.**

9. **Keel S EJ, Block S, Bourne R, Calonge M, Cheng C-Y, Friedman DS, FurtadoJM, Khanna RC, Mathenge W, Mariotti S, Matoto E, Müller A, Rabiu MM, Rasengane T, Zhao J, Wormald R, Cieza A: Strengthening the integration of eye care into the health system: methodology for the development of the WHO package of eye care interventions. *BMJ Open Ophthalmology* 2020, 5(1).**

Acknowledgements

Cochrane Eyes and Vision Steering committee

- Michael Bowen (College of Optometrists)
- Roxanne Crosby-Nwaobi (Moorfields Eye Hospital)
- Jennifer Evans (Co-ordinating Editor, UK editorial base)
- Barny Foot (Royal College of Ophthalmologists)
- Stephen Gichuhi (University of Nairobi)
- Renata Gomes (Blind Veterans)
- John Lawrenson (City University)
- Tianjing Li (Co-ordinating Editor, CEV@US)

For peer review only - http://bmjopen.bmj.com/site/about/guidelines.xhtml

- Jacqui Ramke (Editor with special interest in Equity)
- Fiona Rowe (Liverpool University)
- Anupa Shah (Managing Editor, UK editorial base)
- Gianni Virgili (Co-ordinating Editor, DTA satellite)
- Richard Wormald (Co-ordinating Editor, UK editorial base)

Author statement

All authors contributed to the conception and design of the study and revised it critically for important intellectual content and approved the version of the work to be published. Jennifer Evans wrote the first draft.

Funding statement

Cochrane Eyes and Vision is supported by the National Institute for Health Research (NIHR) Cochrane Infrastructure grant NIHR129461. Jacqueline Ramke's appointment at the University of Auckland is funded by the Buchanan Charitable Foundation, New Zealand.

Conflicts of interest

Tianjing Li directs the Cochrane Eyes and Vision US Project, supported by grant UG1EY020522 from the National Eye Institute, National Institutes of Health.

Word Count

2416

For peer review only

For peer review only - http://bmjopen.bmj.com/site/about/guidelines.xhtml

BMJ Open

Identifying priority review questions for Cochrane Eyes and Vision: protocol for a Priority Setting Exercise

Journal:	*BMJ Open*
Manuscript ID	bmjopen-2020-046319.R1
Article Type:	Protocol
Date Submitted by the Author:	09-May-2021
Complete List of Authors:	Evans, Jennifer; Queen's University Belfast, Centre for Public Health; London School of Hygiene and Tropical Medicine International Centre for Eye Health<br>Gordon, Iris; Queen's University Belfast, Centre for Public Health; London School of Hygiene & Tropical Medicine, International Centre for Eye Health<br>Lawrenson, John; City University of London Division of Optometry and Visual Science<br>Parker, Roses; Oxford University Hospitals NHS Trust, Cochrane MOSS Network<br>Rowe, Fiona; University of Liverpool Department of Health Services Research, Health Services Research<br>Virgili, Gianni; Queen's University Belfast, Centre for Public Health; University of Florence Department of Surgery and Translational Medicine<br>Li, Tianjing; University of Colorado, Anschutz Medical Campus<br>Ramke, Jacqueline; London School of Hygiene & Tropical Medicine, International Centre for Eye Health; The University of Auckland, School of Optometry and Vision Science
**Primary Subject Heading**:	Ophthalmology
Secondary Subject Heading:	Research methods
Keywords:	Medical ophthalmology < OPHTHALMOLOGY, Ophthalmology < SURGERY, Glaucoma < OPHTHALMOLOGY, Cataract and refractive surgery < OPHTHALMOLOGY

SCHOLARONE™
Manuscripts

I, the Submitting Author has the right to grant and does grant on behalf of all authors of the Work (as defined in the below author licence), an exclusive licence and/or a non-exclusive licence for contributions from authors who are: i) UK Crown employees; ii) where BMJ has agreed a CC-BY licence shall apply, and/or iii) in accordance with the terms applicable for US Federal Government officers or employees acting as part of their official duties; on a worldwide, perpetual, irrevocable, royalty-free basis to BMJ Publishing Group Ltd ("BMJ") its licensees and where the relevant Journal is co-owned by BMJ to the co-owners of the Journal, to publish the Work in this journal and any other BMJ products and to exploit all rights, as set out in our licence.

The Submitting Author accepts and understands that any supply made under these terms is made by BMJ to the Submitting Author unless you are acting as an employee on behalf of your employer or a postgraduate student of an affiliated institution which is paying any applicable article publishing charge ("APC") for Open Access articles. Where the Submitting Author wishes to make the Work available on an Open Access basis (and intends to pay the relevant APC), the terms of reuse of such Open Access shall be governed by a Creative Commons licence – details of these licences and which Creative Commons licence will apply to this Work are set out in our licence referred to above.

Other than as permitted in any relevant BMJ Author's Self Archiving Policies, I confirm this Work has not been accepted for publication elsewhere, is not being considered for publication elsewhere and does not duplicate material already published. I confirm all authors consent to publication of this Work and authorise the granting of this licence.

For peer review only

Identifying priority review questions for Cochrane Eyes and Vision: protocol for a Priority Setting Exercise

Jennifer R Evans[1,2], Iris Gordon[1,2], John Lawrenson[3], Roses Parker[4], Fiona J Rowe[5], Gianni Virgili[1,,6], Tianjing Li[7] , Jacqueline Ramke[2,8] and the Cochrane Eyes and Vision Priority Setting Steering Group.

1 Centre for Public Health, Queen's University, Belfast, UK

2 International Centre for Eye Health, London School of Hygiene & Tropical Medicine, London, UK

3 City University, London, UK

4 Cochrane MOSS Network, c/o Oxford University Hospitals NHS Foundation Trust, UK

5 University of Liverpool, UK

6 University of Florence, Italy

7 University of Colorado Anschutz Medical Campus, US

8 School of Optometry and Vision Science, University of Auckland, New Zealand, New Zealand

Corresponding author: Jennifer Evans. International Centre for Eye Health, Department of Clinical Research, London School of Hygiene & Tropical Medicine, Keppel Street, London WC1E 7HT. Jennifer.evans@qub.ac.uk 07789674043

Keywords

Eye, Vision, Priority Setting Exercise, Systematic Review, Cochrane

For peer review only

Abstract

Introduction

Cochrane Eyes and Vision (CEV) is an international network of individuals working to prepare, maintain and promote access to systematic reviews of interventions to treat, prevent or diagnose eye diseases or vision impairment. CEV plans to undertake a priority setting exercise to identify systematically research questions relevant to our scope, and to formally incorporate input from a wide range of stakeholders to set priorities for new and updated reviews.

Methods and analysis

The scope of CEV is broad and our reviews include conditions that are common and have a high global disease burden, for example cataract and dry eye disease, and conditions that are rare but have a high impact on quality of life and high individual cost such as eye cancer. We plan to focus on conditions prioritised by the World Health Organization (WHO) during the development of the Package of Eye Care Interventions. These conditions were selected based on a combination of data on disease magnitude, health care use, and expert opinion. We will identify priority review questions systematically by summarising relevant data on research in Eyes and Vision from a range of sources, and compiling a list of 10-15 potential review questions (new and/or updates for each condition group. We will seek the views of external and internal stakeholders on this list by conducting an online survey. Equity will be a specific consideration.

Ethics and dissemination

The study has been approved by the ethics committee of the London School of Hygiene & Tropical Medicine (LSHTM). We will disseminate the findings through Cochrane channels and prepare a summary of the work for publication in a peer-reviewed journal.

Article summary

Strengths and limitations of this study:

- This is a systematic assessment of priority questions for Cochrane Eyes and Vision systematic reviews.
- We will seek global input, considering questions irrespective of location or setting.
- The study will draw upon a wide range of stakeholders and resources.
- The focus will be mainly on new intervention reviews and topics but we will also consider potential review updates.
- The conduct of an online survey will limit the amount of discussion possible.

Introduction

Cochrane Eyes and Vision (CEV) is an international network of individuals working to prepare, maintain and promote access to systematic reviews of interventions to treat or prevent eye diseases or vision impairment. We also conduct systematic reviews of the accuracy of diagnostic tests for common ocular diseases or conditions.

For peer review only - http://bmjopen.bmj.com/site/about/guidelines.xhtml

CEV was established in 1997.[1] We have used a variety of priority setting approaches over the years, including working with guideline panels [2, 3] and participating in a James Lind Alliance Priority Setting Partnership for Sight Loss and Vision,[4] but we have largely relied on review author teams to suggest review titles. These titles are evaluated by the editorial base and our network of editors (see https://eyes.cochrane.org/about-cev for list) to assess whether they would form a suitable review question, ensuring no overlap with current Cochrane systematic reviews or with high quality, recently published, non-Cochrane systematic reviews. We consider the following criteria when prioritising review titles suggested by review author teams:

- *Does the proposed new review (or review update) address an important clinical uncertainty?* By "important" we mean that the review topic is one that patients, clinicians, policymakers or the public would like to have answered i.e. is important to them. A "clinical uncertainty" reflects the situation where there is evidence of variation in practice or differing opinions as to the best or most effective intervention.
- *Will a Cochrane Review (new review or review update) at this point in time resolve this clinical uncertainty?* Largely this means that we aim to prioritise reviews that will include a number of reasonably large and robust studies. However, sometimes we judge that identifying a gap in the evidence is also important, if identifying that gap will be likely to lead to further intervention studies.
- *To what extent would resolving this clinical uncertainty reduce the magnitude of vision impairment and eye health disorders?* This is clearly a subjective judgement but takes into account knowledge of the magnitude of the eye disease or vision impairment and anticipated effect, or cost-effectiveness, of the intervention.
- *To what extent would resolving this clinical uncertainty reduce inequalities/disparities in the magnitude of disease or access to care for vision impairment or eye health disorders?* Ideally, we would prioritise reviews and interventions that address inequity.

This approach identifies titles that are important to clinicians and researchers but may be less likely to identify questions relevant to other stakeholders such as patients, public and policy makers. It is also likely to result in focus on higher-income settings, with high research capacity, and less emphasis on equity-relevant titles. A recent review highlighted the lack of equity-relevant Cochrane Reviews on cataract .[5]

CEV plans to undertake a priority setting exercise to assess systematically the nature and extent of research questions relevant to our scope, and to formally incorporate input from a wide range of stakeholders to set priorities for new and updated reviews. This document outlines the protocol for a priority setting exercise to identify important review questions. It is informed by guidance prepared by Cochrane on setting review priorities [6] and by the REporting guideline for PRIority SEtting of health research (REPRISE) framework for reporting priority setting of health research. [7] Equity will be a specific consideration in the priority setting process. As part of this process, we will also be informed by the work of other organisations undertaking priority setting activities in the field of Eyes and Vision, including the recent Grand Challenges in global eye health undertaken as part of the *Lancet Global Health* Commission on Global Eye Health[8] and the development of the package of eye care interventions being developed by WHO. [9, 10]

For peer review only - http://bmjopen.bmj.com/site/about/guidelines.xhtml

Aims

The CEV Priority Setting Exercise aims to generate and publicise a list of priority topics, for both new and updated reviews, ensuring involvement of our main stakeholders.

Methods

Context and scope

The scope of CEV is global and includes conditions that are common and have a high global disease burden, for example cataract and dry eye disease, and conditions that are rare but have a high impact on quality of life and high individual cost such as eye cancer. The intended beneficiaries of our work are people making health-care decisions for eye health care. CEV reviews primarily address questions relevant to clinical research (treatment, diagnosis. prognosis) but also potentially cover public health, health services and implementation research.

We plan to focus on the list set out in *Box 1* adapted from conditions prioritised by WHO as part of the development of the Package of Eye Care Interventions (PECI). These conditions have been selected based on a combination of data on disease magnitude, health care use, and expert opinion.

Box 1: List of conditions

1. Cataract
2. Refractive error
3. Diabetic retinopathy
4. Glaucoma
5. Macular degeneration
6. Amblyopia
7. Disorders of eye movement
8. Infectious and inflammatory diseases
9. Ocular trauma
10. Ocular surface disorders
11. Disorders of the eyelid and lacrimal system
12. Eye cancer

Governance

To advise on the scope of the exercise, we set up a Steering Group including ophthalmologists, optometrists, orthoptists, ophthalmic nurses and relevant professional bodies, patient organisations, experienced clinical editors, systematic review methodologists and information specialists of CEV; it includes participants from high and low-income settings. Members of the team have been involved in previous priority setting exercises.

Stakeholders and participants

Any person with an interest in health-care decision-making relevant to eye health care is eligible to take part. This includes patients, caregivers, the general public, health professions, researchers, policy makers, government and non-government organisations, and industry. We will actively seek out potential stakeholders using the expertise of the Steering Group to identify relevant stakeholder organisations and individuals globally in their field. We will then write to each stakeholder

For peer review only - http://bmjopen.bmj.com/site/about/guidelines.xhtml

organisation or individual identified by the Steering Group to invite them to take part in the online survey and to identify further relevant stakeholders (snowballing).

Identifying research questions

For each condition we will undertake the following steps in order to identify priority questions for each condition, consulting with the Steering Group as needed (figure 1):

(1) Identify and summarise relevant data on research in Eyes and Vision. We plan to use the following sources:

- Global policy reports
- Other research prioritisation and roadmaps
- Guidelines
- CEV systematic reviews
- CEV@US Project Database of Systematic Reviews in Eyes and Vision [11]
- Intervention studies on *The Cochrane Library* (CENTRAL)

We will identify global policy reports and other research prioritisation exercises (for example, James Lind Alliance Priority Setting Process in Sight Loss and Vision[4]) from searching academic databases and contact with our Steering Group. One of members of the research team will scrutinise these reports for identified research questions. We will identify high-quality guidelines from the process followed by the WHO during the development of the Package of Eye Care Interventions. We will rank current CEV reviews according to impact using Altmetric and access statistics provided by the publisher. The top 20% of reviews identified by this process i.e. reviews with highest Altmetric score and/or access statistics will be eligible for the priority setting process. We will search *The Cochrane Library* for studies relevant to Eyes and Vision published within the last three years, or on a trials registers. One author (IG) will scan these reports to identify new potential review titles i.e. PICOs identified in two or more studies, that have not already been addressed in Cochrane Reviews. Search strategies are in appendix 1.

(2) Using the information from (1) we will prepare a list of potential review topics, removing duplicates as needed or merging/combining similar questions. This list will be reviewed by the Steering Group after it has been refined by removing questions where:

- High quality, current systematic reviews exist and new trials are either unlikely to have been done, or if they have been done, are unlikely to change the conclusions of the review. We will check for Cochrane Reviews and non-Cochrane high quality systematic reviews in the database maintained by CEV@US project.
- Topic is beyond scope because either it does not address a condition of interest (*Box 1*) or it does not address a relevant clinical question.
- Question is unclear or ill-defined or cannot be answered by either an intervention, diagnostic test accuracy, prognostic or scoping review.

For peer review only - http://bmjopen.bmj.com/site/about/guidelines.xhtml

Conducting the priority setting exercise

We will seek the views of external and internal stakeholders on the list generated in (2) by conducting an online survey using Qualtrics software (Qualtrics, Provo, Utah USA available at www.qualtrics.com).

We will identify key stakeholders, initially by consultation with the Steering Group, but also including further "snowballing" I.e. asking stakeholders to identify other relevant stakeholders. We will request information on key research needs. The categories of stakeholders that we will consider include but will not be restricted to:

- Patients and public
- Providers, purchasers and payers
- Policy makers
- Principal investigators and research funder

Participants will be identified through two methods: advertising and direct invitation. We will advertise through social media (e.g., Twitter, Facebook, and Cochrane channels) and within academic (e.g., Alumni networks, Community Eye Health Bulletin,) clinical and professional networks (e.g., ophthalmological, optometric and orthoptic societies). We will inform the stakeholders identified above, and ask members of the Steering Group, to circulate within their networks. We will invite some participants directly to take part and will ensure that these invitations are balanced with respect to gender, location (working/living in a high-income or lower-income setting) and profession (clinician, patient, policymaker). We will approach Cochrane contributors and other contacts we consider potentially interested to contribute and will draw upon the previous participants of the Delphi exercise for Grand Challenges for Eye Health (names available in public domain). We will not perform any other formal process for ensuring balance, but we will collect limited information on respondents (gender, location, profession) and how they were informed about the survey so that we understand who has responded.

We will conduct a two-round process. In the first round, we will present questions separately for each condition and ask the participants to rank in order of priority. There will be space for the participant to add additional questions that have not been included in the presented list. We will be seeking questions relevant to interventions reviews, diagnostic test accuracy reviews, prognostic reviews, or scoping reviews only. Within 4 weeks we will present a second round, in which the top five questions for each condition will be presented, along with any additional new questions identified during the course of the first round. Participants will be asked to score the review questions according to the following criteria (4-point scale 1=definitely not, 2=possibly not, 3=possibly yes, 4=definitely yes):

- Does the proposed new review (or review update) address an important clinical uncertainty?
- Will a Cochrane Review (new review or review update) at this point in time resolve this clinical uncertainty?
- To what extent would resolving this clinical uncertainty reduce the magnitude of vision impairment and eye health disorders?

For peer review only - http://bmjopen.bmj.com/site/about/guidelines.xhtml

- To what extent would resolving this clinical uncertainty reduce inequalities/disparities in (i.e. have an equalizing effect of) the magnitude of disease or access to care for vision impairment or eye health disorders?

Following these two rounds, for each condition, we will identify the three questions with the highest average score. Each question will be structured in the PICO format, adapted for other contexts for example diagnosis, prognosis as necessary. As part of this process, we will be guided by the quantitative and qualitative results of these surveys but, to align with our commitment to widen the inclusion and equity-relevance of CEV reviews, we will include at least one question relevant to lower income settings. We will also report ranking of review question priorities by location and stakeholder background to assess the extent to which priorities within different groups differ.

Equity

We will consider equity as part of this process, drawing upon methods developed by the Campbell and Cochrane Equity Methods group (https://methods.cochrane.org/equity/about-us). One member of the Steering Group has a special interest in equity. Our main approach will be to ensure that we have as wide a participation as possible (see above for details). We are taking the opportunity to draw upon current partnerships with global initiatives to ensure priorities are informed by representation from low- and middle- income countries. We will also prioritise questions that the survey participants have considered would reduce inequalities (last question above).

Patient and public involvement

Patient and public involvement will be through the Steering Group and by contributing to the priority setting exercise.

Ethics and dissemination

The study has been approved by the ethics committee of the LSHTM. Please see appendix 2 for information to be given to participants in the online survey. All data collection will be electronic. We will disseminate the findings through Cochrane channels and prepare a summary of the work for publication in a peer-reviewed journal.

We will publish, through relevant Cochrane channels, our intention to conduct a priority setting process so that external and internal stakeholders may be involved.

We plan to:

- Document our plans for priority setting including stakeholder engagement, methods and criteria.
- Document the implementation of the priority-setting process on our website (including link to relevant network portal) and in an academic publication.
- Publish a list of priority topics on the CEV website.
- Develop a plan for how the priority topics will be delivered.
- Provide feedback to stakeholders involved, including notification when priority reviews are published.

For peer review only - http://bmjopen.bmj.com/site/about/guidelines.xhtml
For peer review only

Currency/timeframe

We plan to complete the Priority Setting Exercise during June to December 2021 and repeat within 3 to 5 years.

Evaluation and feedback

Written feedback will be given to all participants in the process who have supplied an email address, including a plain language account of the process and outcome of the process. All participants will be acknowledged in the final report (with permission).

We will evaluate the priority setting process by asking participants to complete a questionnaire collecting quantitative data and qualitative information on the following outcomes.

<u>Short-term evaluation</u>

1. Did the priority setting process meet Cochrane mandatory and desirable criteria for governance, stakeholder engagement, documentation and dissemination?
2. Was the process complete within the pre-specified time frame?
3. Was the process completed without using excessive CEV staff time?
4. Gather feedback from stakeholders via questionnaire
    a. What did stakeholders like about the process?
    b. What did stakeholders want to improve about the process?
5. Gather feedback from CEV staff
    a. What did CEV staff like about the process?
    b. What did CEV staff want to improve about the process?

<u>Long-term evaluation</u>

1. Were the resultant reviews produced in a timely manner?
2. Were the resultant reviews relevant/important? For example, did they have higher Altmetric/impact score?
3. Were the reviews used in guidelines or other policy documents?

Other considerations:

- Equity – how have the results improved equity? Have any of the reviews considered most relevant for equity in the process above been undertaken?
- Has there been an increase in authors from low- and middle-income settings?

Conclusion

A systematic and transparent approach to identifying review questions, informed by credible evidence, and reaching out to a broader group of people to assess priorities will help CEV establish which reviews need to be prioritised in the next 3 to 5 years.

For peer review only - http://bmjopen.bmj.com/site/about/guidelines.xhtml

References

1. Evans J, Li T, Virgili G, Wormald R, Cochrane E, Vision: Cochrane Eyes and Vision: a perspective introducing Cochrane Corner in Eye. *Eye* 2019, 33(6):882-886.

2. NICE: Age-related macular degeneration: diagnosis and management In: *NICE Guideline NG82.* National Institute of Health and Care Excellence; 2018.

3. NICE: Cataracts in adults: management. In: *NICE Guideline NG77.* National Institute of Health and Care Excellence; 2017.

4. Rowe F, Wormald R, Cable R, Acton M, Bonstein K, Bowen M, Bronze C, Bunce C, Conroy D, Cowan K *et al*: The Sight Loss and Vision Priority Setting Partnership (SLV-PSP): overview and results of the research prioritisation survey process. *BMJ open* 2014, 4(7):e004905.

5. Evans J, Mwangi N, Burn H, Ramke J: Equity was rarely considered in Cochrane Eyes and Vision systematic reviews and primary studies on cataract. *Journal of clinical epidemiology* 2020, 125:57-63.

6. Cochrane: Guidance note for Cochrane Groups to define systematic review priorities. In.; 2019.

7. Tong A, Synnot A, Crowe S, Hill S, Matus A, Scholes-Robertson N, Oliver S, Cowan K, Nasser M, Bhaumik S *et al*: Reporting guideline for priority setting of health research (REPRISE). *BMC medical research methodology* 2019, 19(1):243.

8. Burton MJ, Ramke J, Marques AP, Bourne RRA, Congdon N, Jones I, Ah Tong BAM, Arunga S, Bachani D, Bascaran C *et al*: The Lancet Global Health Commission on Global Eye Health: vision beyond 2020. *The Lancet Global health* 2021, 9(4):e489-e551.

9. WHO: World report on vision. . In. Geneva: World Health Organization

2019.

10. Keel S EJ, Block S, Bourne R, Calonge M, Cheng C-Y, Friedman DS, FurtadoJM, Khanna RC, Mathenge W, Mariotti S, Matoto E, Müller A, Rabiu MM, Rasengane T, Zhao J, Wormald R, Cieza A: Strengthening the integration of eye care into the health system: methodology for the development of the WHO package of eye care interventions. *BMJ Open Ophthalmology* 2020, 5(1).

11. Le J QR, Rouse B, Twose C, Rosman L, Lindsley K, Hawkins BS, Li T. : Development and content of a database of systematic reviews for eyes and vision. . *Eye* 2021, in press.

Acknowledgements

Cochrane Eyes and Vision Steering committee

- Michael Bowen (College of Optometrists)

For peer review only - http://bmjopen.bmj.com/site/about/guidelines.xhtml

- Roxanne Crosby-Nwaobi (Moorfields Eye Hospital)
- Jennifer Evans (Co-ordinating Editor, UK editorial base)
- Barny Foot (Royal College of Ophthalmologists)
- Stephen Gichuhi (University of Nairobi)
- Renata Gomes (Blind Veterans)
- John Lawrenson (City University)
- Tianjing Li (Co-ordinating Editor, CEV@US)
- Roses Parker (Cochrane Network Support Fellow)
- Jacqui Ramke (Editor with special interest in Equity)
- Fiona Rowe (Liverpool University)
- Anupa Shah (Managing Editor, UK editorial base)
- Gianni Virgili (Co-ordinating Editor, DTA satellite)
- Richard Wormald (Co-ordinating Editor, UK editorial base)

Author statement

All authors contributed to the conception and design of the study through participation in the Steering Group. JE wrote the first draft of the paper. JL, RP, FR GV, TL, JR revised it critically for important intellectual content. IG prepared the search strategies. All authors approved the submitted paper. As this is a protocol, acquisition, analysis and interpretation of data do not apply.

Funding statement

Cochrane Eyes and Vision is supported by the National Institute for Health Research (NIHR) Cochrane Infrastructure grant NIHR129461 until end March 2021 and Northern Ireland Health and Social Care Agency from April 1st 2021. Jacqueline Ramke's appointment at the University of Auckland is funded by the Buchanan Charitable Foundation, New Zealand.

Conflicts of interest

Tianjing Li directs the Cochrane Eyes and Vision US Project, supported by grant UG1EY020522 from the National Eye Institute, National Institutes of Health.

Word Count

2517

For peer review only - http://bmjopen.bmj.com/site/about/guidelines.xhtml

Figure 1: Flow-chart of study process

For peer review only

For peer review only - http://bmjopen.bmj.com/site/about/guidelines.xhtml

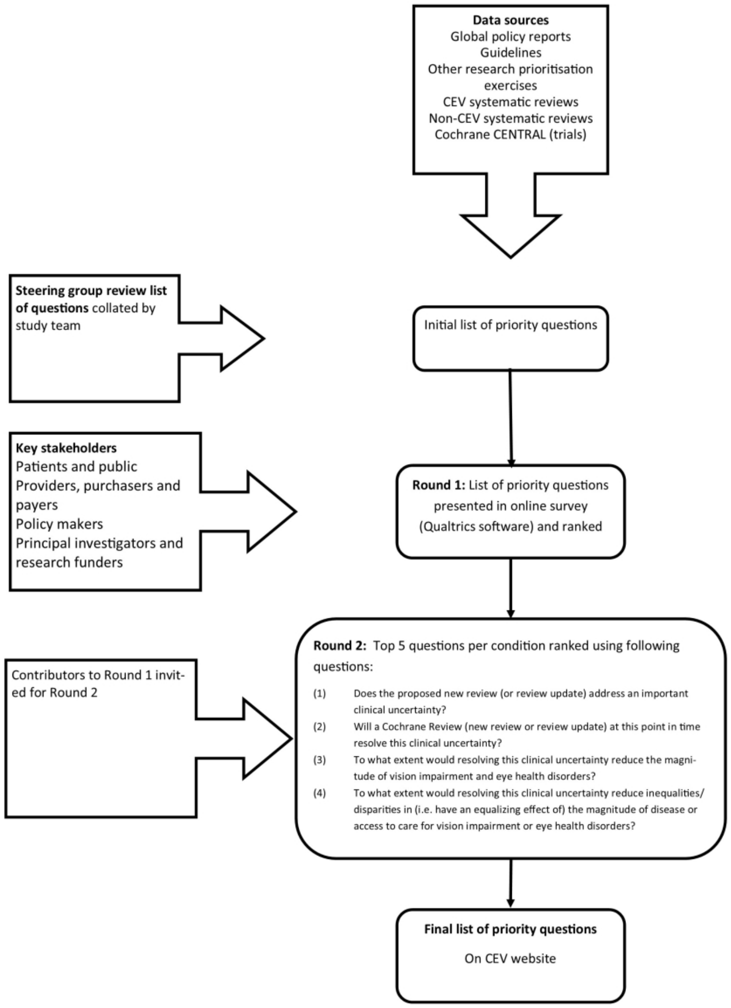

Figure 1: Flow-chart of study process

209x297mm (300 x 300 DPI)

Appendix 1: Search strategies

Roadmaps and other priority setting exercises (Google)

- roadmap ophthalmology
- unmet need ophthalmology
- priority setting ophthalmology
- prioritization research ophthalmology
- roadmap eye
- unmet need eye
- priority setting eye
- prioritization research eye

Cochrane Central register of controlled trials (CENTRAL)

- exp cataract and exp cataract extraction
- exp amblyopia/
- Strabismus/
- Ocular Motility Disorders/
- exp Nystagmus, Pathologic
- exp diabetic retinopathy
- exp eyelid diseases/
- Lacrimal Apparatus Diseases/
- Lacrimal Duct Obstruction/
- Nasolacrimal Duct/
- exp eye neoplasms
- exp glaucoma
- exp conjunctivitis/
- exp keratitis/
- exp uveitis/
- exp endophthalmitis/
- exp dry eye syndromes
- exp refractive errors
- vision, low
- visually impaired persons”
- blindness
- exp macular degeneration

For peer review only

For peer review only - http://bmjopen.bmj.com/site/about/guidelines.xhtml

Appendix 2: Information for participants / online survey

Cochrane Eyes and Vision Priority Setting Exercise

You are being invited to take part in a research study. Before you decide to take part please read the following information. If you are happy to participate, please tick the box below. Please contact Cochrane Eyes and Vision <email> if there is anything that is not clear or if you would like more information.

Purpose of the study: Cochrane Eyes and Vision plans to undertake a priority setting exercise to assess systematically the nature and extent of research questions relevant to our scope, and to formally incorporate input from a wider range of stakeholders to set priorities for new and updated reviews.

What is involved in taking part in the study? We would like you to take part in two rounds by online questionnaire. This is the first round and we expect it will take around 10 minutes to do. On the following pages, you will be presented with a list of potential review topics relevant to refractive error and asked to rank them in order of importance. These potential review topics were identified by systematic searching of global policy reports, guidelines and reports of relevant reviews and studies. We may have missed important questions and so there will also be an opportunity to tell us of priority topics that are not on the list.

Different eye conditions are considered separately and there are separate surveys available for other eye conditions (cataract, glaucoma etc).

Participants who provide their email address will be invited to take part in the second round. In the second round, you will be asked to rank the identified top priority topics according to four criteria. Based on responses to this survey, a final list with the highest ranked top 3 priority topics for each condition will be created.

Confidentiality: All information collected during the course of the research will be kept strictly confidential and will not be shared with anyone outside the research team. This information will be destroyed at the conclusion of the project. Your identity may be known to the researchers if you supply an identifiable email. All responses will be de-identified prior to analysis, and results will be reported at an aggregate level, so that your responses will not be identifiable as belonging to you.

What are the benefits? Participating in this study provides the opportunity to identify priorities for Cochrane Eyes and Vision reviews. These priorities may help guide eye health research and funding in future. Cochrane Eyes and Vision will prioritise the conduct of reviews on topics identified in this process. A summary of this priority setting exercise and the final list of priority topics will be published on Cochrane Eyes and Vision website and through relevant Cochrane channels, as well as in a scientific journal. If you provide your email address, we will also send you this information by email.

What are the risks? There are no risks of physical or psychological harm associated with this process. This first round is anticipated to take 10 minutes of your time at this stage, for each eye condition, and then the subsequent round will take approximately 15 minutes. You will not receive financial or

For peer review only - http://bmjopen.bmj.com/site/about/guidelines.xhtml

For peer review only

other type of reimbursement for taking part in the study. Participants who take part in both rounds will be acknowledged as part of group authorship, if they wish.

There is no obligation to take part, and if you do agree to take part you are still free to withdraw at any time and without giving a reason. This study has gained ethical approval from the London School of Hygiene and Tropical Medicine Research Ethics Committee.

Consent: I have read the information above and by ticking this box I consent to be involved in this study. I understand that at any time I may withdraw from this study without give a reason.

For peer review only

BMJ Open

Identifying priority review questions for Cochrane Eyes and Vision: protocol for a Priority Setting Exercise

Journal:	*BMJ Open*
Manuscript ID	bmjopen-2020-046319.R2
Article Type:	Protocol
Date Submitted by the Author:	01-Jun-2021
Complete List of Authors:	Evans, Jennifer; Queen's University Belfast, Centre for Public Health; London School of Hygiene and Tropical Medicine International Centre for Eye Health<br>Gordon, Iris; Queen's University Belfast, Centre for Public Health; London School of Hygiene & Tropical Medicine, International Centre for Eye Health<br>Lawrenson, John; City University of London Division of Optometry and Visual Science<br>Parker, Roses; Oxford University Hospitals NHS Trust, Cochrane MOSS Network<br>Rowe, Fiona; University of Liverpool Department of Health Services Research, Health Services Research<br>Virgili, Gianni; Queen's University Belfast, Centre for Public Health; University of Florence Department of Surgery and Translational Medicine<br>Li, Tianjing; University of Colorado, Anschutz Medical Campus<br>Ramke, Jacqueline; London School of Hygiene & Tropical Medicine, International Centre for Eye Health; The University of Auckland, School of Optometry and Vision Science
**Primary Subject Heading**:	Ophthalmology
Secondary Subject Heading:	Research methods
Keywords:	Medical ophthalmology < OPHTHALMOLOGY, Ophthalmology < SURGERY, Glaucoma < OPHTHALMOLOGY, Cataract and refractive surgery < OPHTHALMOLOGY

SCHOLARONE™
Manuscripts

I, the Submitting Author has the right to grant and does grant on behalf of all authors of the Work (as defined in the below author licence), an exclusive licence and/or a non-exclusive licence for contributions from authors who are: i) UK Crown employees; ii) where BMJ has agreed a CC-BY licence shall apply, and/or iii) in accordance with the terms applicable for US Federal Government officers or employees acting as part of their official duties; on a worldwide, perpetual, irrevocable, royalty-free basis to BMJ Publishing Group Ltd ("BMJ") its licensees and where the relevant Journal is co-owned by BMJ to the co-owners of the Journal, to publish the Work in this journal and any other BMJ products and to exploit all rights, as set out in our licence.

The Submitting Author accepts and understands that any supply made under these terms is made by BMJ to the Submitting Author unless you are acting as an employee on behalf of your employer or a postgraduate student of an affiliated institution which is paying any applicable article publishing charge ("APC") for Open Access articles. Where the Submitting Author wishes to make the Work available on an Open Access basis (and intends to pay the relevant APC), the terms of reuse of such Open Access shall be governed by a Creative Commons licence – details of these licences and which Creative Commons licence will apply to this Work are set out in our licence referred to above.

Other than as permitted in any relevant BMJ Author's Self Archiving Policies, I confirm this Work has not been accepted for publication elsewhere, is not being considered for publication elsewhere and does not duplicate material already published. I confirm all authors consent to publication of this Work and authorise the granting of this licence.

For peer review only

Identifying priority review questions for Cochrane Eyes and Vision: protocol for a Priority Setting Exercise

Jennifer R Evans[1,2], Iris Gordon[1,2], John Lawrenson[3], Roses Parker[4], Fiona J Rowe[5], Gianni Virgili[1,,6], Tianjing Li[7] , Jacqueline Ramke[2,8] and the Cochrane Eyes and Vision Priority Setting Steering Group.

1 Centre for Public Health, Queen's University, Belfast, UK

2 International Centre for Eye Health, London School of Hygiene & Tropical Medicine, London, UK

3 City University, London, UK

4 Cochrane MOSS Network, c/o Oxford University Hospitals NHS Foundation Trust, UK

5 University of Liverpool, UK

6 University of Florence, Italy

7 University of Colorado Anschutz Medical Campus, US

8 School of Optometry and Vision Science, University of Auckland, New Zealand, New Zealand

Corresponding author: Jennifer Evans. International Centre for Eye Health, Department of Clinical Research, London School of Hygiene & Tropical Medicine, Keppel Street, London WC1E 7HT. Jennifer.evans@qub.ac.uk 07789674043

Keywords

Eye, Vision, Priority Setting Exercise, Systematic Review, Cochrane

For peer review only

For peer review only - http://bmjopen.bmj.com/site/about/guidelines.xhtml

Abstract

Introduction

Cochrane Eyes and Vision (CEV) is an international network of individuals working to prepare, maintain and promote access to systematic reviews of interventions to treat, prevent or diagnose eye diseases or vision impairment. CEV plans to undertake a priority setting exercise to identify systematically research questions relevant to our scope, and to formally incorporate input from a wide range of stakeholders to set priorities for new and updated reviews.

Methods and analysis

The scope of CEV is broad and our reviews include conditions that are common and have a high global disease burden, for example cataract and dry eye disease, and conditions that are rare but have a high impact on quality of life and high individual cost such as eye cancer. We plan to focus on conditions prioritised by the World Health Organization (WHO) during the development of the Package of Eye Care Interventions. These conditions were selected based on a combination of data on disease magnitude, health care use, and expert opinion. We will identify priority review questions systematically by summarising relevant data on research in Eyes and Vision from a range of sources, and compiling a list of 10-15 potential review questions (new and/or updates for each condition group. We will seek the views of external and internal stakeholders on this list by conducting an online survey. Equity will be a specific consideration.

Ethics and dissemination

The study has been approved by the ethics committee of the London School of Hygiene & Tropical Medicine (LSHTM). We will disseminate the findings through Cochrane channels and prepare a summary of the work for publication in a peer-reviewed journal.

Article summary

Strengths and limitations of this study:

- This is a systematic assessment of priority questions for Cochrane Eyes and Vision systematic reviews.
- We will seek global input, considering questions irrespective of location or setting.
- The study will draw upon a wide range of stakeholders and resources.
- The focus will be mainly on new intervention reviews and topics but we will also consider potential review updates.
- The conduct of an online survey will limit the amount of discussion possible.

Introduction

Cochrane Eyes and Vision (CEV) is an international network of individuals working to prepare, maintain and promote access to systematic reviews of interventions to treat or prevent eye diseases or vision impairment. We also conduct systematic reviews of the accuracy of diagnostic tests for common ocular diseases or conditions.

For peer review only - http://bmjopen.bmj.com/site/about/guidelines.xhtml

CEV was established in 1997.[1] We have used a variety of priority setting approaches over the years, including working with guideline panels [2, 3] and participating in a James Lind Alliance Priority Setting Partnership for Sight Loss and Vision,[4] but we have largely relied on review author teams to suggest review titles. These titles are evaluated by the editorial base and our network of editors (see https://eyes.cochrane.org/about-cev for list) to assess whether they would form a suitable review question, ensuring no overlap with current Cochrane systematic reviews or with high quality, recently published, non-Cochrane systematic reviews. We consider the following criteria when prioritising review titles suggested by review author teams:

- *Does the proposed new review (or review update) address an important clinical uncertainty?* By “important” we mean that the review topic is one that patients, clinicians, policymakers or the public would like to have answered i.e. is important to them. A “clinical uncertainty” reflects the situation where there is evidence of variation in practice or differing opinions as to the best or most effective intervention.
- *Will a Cochrane Review (new review or review update) at this point in time resolve this clinical uncertainty?* Largely this means that we aim to prioritise reviews that will include a number of reasonably large and robust studies. However, sometimes we judge that identifying a gap in the evidence is also important, if identifying that gap will be likely to lead to further intervention studies.
- *To what extent would resolving this clinical uncertainty reduce the magnitude of vision impairment and eye health disorders?* This is clearly a subjective judgement but takes into account knowledge of the magnitude of the eye disease or vision impairment and anticipated effect, or cost-effectiveness, of the intervention.
- *To what extent would resolving this clinical uncertainty reduce inequalities/disparities in the magnitude of disease or access to care for vision impairment or eye health disorders?* Ideally, we would prioritise reviews and interventions that address inequity.

This approach identifies titles that are important to clinicians and researchers but may be less likely to identify questions relevant to other stakeholders such as patients, public and policy makers. It is also likely to result in focus on higher-income settings, with high research capacity, and less emphasis on equity-relevant titles. A recent review highlighted the lack of equity-relevant Cochrane Reviews on cataract .[5]

CEV plans to undertake a priority setting exercise to assess systematically the nature and extent of research questions relevant to our scope, and to formally incorporate input from a wide range of stakeholders to set priorities for new and updated reviews. This document outlines the protocol for a priority setting exercise to identify important review questions. It is informed by guidance prepared by Cochrane on setting review priorities [6] and by the REporting guideline for PRIority SEtting of health research (REPRISE) framework for reporting priority setting of health research. [7] Equity will be a specific consideration in the priority setting process. As part of this process, we will also be informed by the work of other organisations undertaking priority setting activities in the field of Eyes and Vision, including the recent Grand Challenges in global eye health undertaken as part of the *Lancet Global Health* Commission on Global Eye Health[8] and the development of the package of eye care interventions being developed by WHO. [9, 10]

For peer review only - http://bmjopen.bmj.com/site/about/guidelines.xhtml

Aims

The CEV Priority Setting Exercise aims to generate and publicise a list of priority topics, for both new and updated reviews, ensuring involvement of our main stakeholders.

Methods

Context and scope

The scope of CEV is global and includes conditions that are common and have a high global disease burden, for example cataract and dry eye disease, and conditions that are rare but have a high impact on quality of life and high individual cost such as eye cancer. The intended beneficiaries of our work are people making health-care decisions for eye health care. CEV reviews primarily address questions relevant to clinical research (treatment, diagnosis. prognosis) but also potentially cover public health, health services and implementation research.

We plan to focus on the list set out in *Box 1* adapted from conditions prioritised by WHO as part of the development of the Package of Eye Care Interventions (PECI). These conditions have been selected based on a combination of data on disease magnitude, health care use, and expert opinion.

Box 1: List of conditions

1. Cataract
2. Refractive error
3. Diabetic retinopathy
4. Glaucoma
5. Macular degeneration
6. Amblyopia
7. Disorders of eye movement
8. Infectious and inflammatory diseases
9. Ocular trauma
10. Ocular surface disorders
11. Disorders of the eyelid and lacrimal system
12. Eye cancer

Governance

To advise on the scope of the exercise, we set up a Steering Group including ophthalmologists, optometrists, orthoptists, ophthalmic nurses and relevant professional bodies, patient organisations, experienced clinical editors, systematic review methodologists and information specialists of CEV; it includes participants from high and low-income settings. Members of the team have been involved in previous priority setting exercises.

Stakeholders and participants

Any person with an interest in health-care decision-making relevant to eye health care is eligible to take part. This includes patients, caregivers, the general public, health professions, researchers, policy makers, government and non-government organisations, and industry. We will actively seek out potential stakeholders using the expertise of the Steering Group to identify relevant stakeholder organisations and individuals globally in their field. We will then write to each stakeholder

For peer review only - http://bmjopen.bmj.com/site/about/guidelines.xhtml

organisation or individual identified by the Steering Group to invite them to take part in the online survey and to identify further relevant stakeholders (snowballing).

Identifying research questions

For each condition we will undertake the following steps in order to identify priority questions for each condition, consulting with the Steering Group as needed (figure 1):

(1) Identify and summarise relevant data on research in Eyes and Vision. We plan to use the following sources:

- Global policy reports
- Other research prioritisation and roadmaps
- Guidelines
- CEV systematic reviews
- CEV@US Project Database of Systematic Reviews in Eyes and Vision [11]
- Intervention studies on *The Cochrane Library* (CENTRAL)

We will identify global policy reports and other research prioritisation exercises (for example, James Lind Alliance Priority Setting Process in Sight Loss and Vision[4]) from searching academic databases and contact with our Steering Group. One of members of the research team will scrutinise these reports for identified research questions. We will identify high-quality guidelines from the process followed by the WHO during the development of the Package of Eye Care Interventions. We will rank current CEV reviews according to impact using Altmetric and access statistics provided by the publisher. The top 20% of reviews identified by this process i.e. reviews with highest Altmetric score and/or access statistics will be eligible for the priority setting process. We will search *The Cochrane Library* for studies relevant to Eyes and Vision published within the last three years, or on a trials registers. One author (IG) will scan these reports to identify new potential review titles i.e. PICOs identified in two or more studies, that have not already been addressed in Cochrane Reviews. Search strategies are in appendix 1.

(2) Using the information from (1) we will prepare a list of potential review topics, removing duplicates as needed or merging/combining similar questions. This list will be reviewed by the Steering Group after it has been refined by removing questions where:

- High quality, current systematic reviews exist and new trials are either unlikely to have been done, or if they have been done, are unlikely to change the conclusions of the review. We will check for Cochrane Reviews and non-Cochrane high quality systematic reviews in the database maintained by CEV@US project.
- Topic is beyond scope because either it does not address a condition of interest (*Box 1*) or it does not address a relevant clinical question.
- Question is unclear or ill-defined or cannot be answered by either an intervention, diagnostic test accuracy, prognostic or scoping review.

For peer review only - http://bmjopen.bmj.com/site/about/guidelines.xhtml

Conducting the priority setting exercise

We will seek the views of external and internal stakeholders on the list generated in (2) by conducting an online survey using Qualtrics software (Qualtrics, Provo, Utah USA available at www.qualtrics.com).

We will identify key stakeholders, initially by consultation with the Steering Group, but also including further "snowballing" I.e. asking stakeholders to identify other relevant stakeholders. We will request information on key research needs. The categories of stakeholders that we will consider include but will not be restricted to:

- Patients and public
- Providers, purchasers and payers
- Policy makers
- Principal investigators and research funder

Participants will be identified through two methods: advertising and direct invitation. We will advertise through social media (e.g., Twitter, Facebook, and Cochrane channels) and within academic (e.g., Alumni networks, Community Eye Health Bulletin,) clinical and professional networks (e.g., ophthalmological, optometric and orthoptic societies). We will inform the stakeholders identified above, and ask members of the Steering Group, to circulate within their networks. We will invite some participants directly to take part and will ensure that these invitations are balanced with respect to gender, location (working/living in a high-income or lower-income setting) and profession (clinician, patient, policymaker). We will approach Cochrane contributors and other contacts we consider potentially interested to contribute and will draw upon the previous participants of the Delphi exercise for Grand Challenges for Eye Health (names available in public domain). We will not perform any other formal process for ensuring balance, but we will collect limited information on respondents (gender, location, profession) and how they were informed about the survey so that we understand who has responded.

We will conduct a two-round process. In the first round, we will present questions separately for each condition and ask the participants to rank in order of priority, that is, which reviews or review updates should Cochrane Eyes and Vision complete first, in the opinion of the respondent. There will be space for the participant to add additional questions that have not been included in the presented list. We will be seeking questions relevant to interventions reviews, diagnostic test accuracy reviews, prognostic reviews, or scoping reviews only. Within 4 weeks we will present a second round, in which the top five questions for each condition will be presented, along with any additional new questions identified during the course of the first round. Participants will be asked to score each review question presented according to the following criteria (4-point scale 1=definitely not/no extent, 2=possibly not/small extent, 3=possibly yes/moderate extent, 4=definitely yes/large extent):

- Does the proposed new review (or review update) address an important clinical uncertainty?
- Will a Cochrane Review (new review or review update) at this point in time resolve this clinical uncertainty?
- To what extent would resolving this clinical uncertainty reduce the magnitude of vision impairment and eye health disorders?

For peer review only - http://bmjopen.bmj.com/site/about/guidelines.xhtml

- To what extent would resolving this clinical uncertainty reduce inequalities/disparities in (i.e. have an equalizing effect of) the magnitude of disease or access to care for vision impairment or eye health disorders?

Following these two rounds, for each condition, we will identify the three questions with the highest average score. Each question will be structured in the PICO format, adapted for other contexts for example diagnosis, prognosis as necessary. As part of this process, we will be guided by the quantitative and qualitative results of these surveys but, to align with our commitment to widen the inclusion and equity-relevance of CEV reviews, we will include at least one question relevant to lower income settings. We will also report ranking of review question priorities by location and stakeholder background to assess the extent to which priorities within different groups differ.

Equity

We will consider equity as part of this process, drawing upon methods developed by the Campbell and Cochrane Equity Methods group (https://methods.cochrane.org/equity/about-us). One member of the Steering Group has a special interest in equity. Our main approach will be to ensure that we have as wide a participation as possible (see above for details). We are taking the opportunity to draw upon current partnerships with global initiatives to ensure priorities are informed by representation from low- and middle- income countries. We will also prioritise questions that the survey participants have considered would reduce inequalities (last question above).

Patient and public involvement

Patient and public involvement will be through the Steering Group and by contributing to the priority setting exercise.

Ethics and dissemination

The study has been approved by the ethics committee of the LSHTM. Please see appendix 2 for information to be given to participants in the online survey. All data collection will be electronic. We will disseminate the findings through Cochrane channels and prepare a summary of the work for publication in a peer-reviewed journal.

We will publish, through relevant Cochrane channels, our intention to conduct a priority setting process so that external and internal stakeholders may be involved.

We plan to:

- Document our plans for priority setting including stakeholder engagement, methods and criteria.
- Document the implementation of the priority-setting process on our website (including link to relevant network portal) and in an academic publication.
- Publish a list of priority topics on the CEV website.
- Develop a plan for how the priority topics will be delivered.
- Provide feedback to stakeholders involved, including notification when priority reviews are published.

For peer review only - http://bmjopen.bmj.com/site/about/guidelines.xhtml

For peer review only

Currency/timeframe

We plan to complete the Priority Setting Exercise during June to December 2021 and repeat within 3 to 5 years.

Evaluation and feedback

Written feedback will be given to all participants in the process who have supplied an email address, including a plain language account of the process and outcome of the process. All participants will be acknowledged in the final report (with permission).

We will evaluate the priority setting process by asking participants to complete a questionnaire collecting quantitative data and qualitative information on the following outcomes.

<u>Short-term evaluation</u>

1. Did the priority setting process meet Cochrane mandatory and desirable criteria for governance, stakeholder engagement, documentation and dissemination?
2. Was the process complete within the pre-specified time frame?
3. Was the process completed without using excessive CEV staff time?
4. Gather feedback from stakeholders via questionnaire
    a. What did stakeholders like about the process?
    b. What did stakeholders want to improve about the process?
5. Gather feedback from CEV staff
    a. What did CEV staff like about the process?
    b. What did CEV staff want to improve about the process?

<u>Long-term evaluation</u>

1. Were the resultant reviews produced in a timely manner?
2. Were the resultant reviews relevant/important? For example, did they have higher Altmetric/impact score?
3. Were the reviews used in guidelines or other policy documents?

Other considerations:

- Equity – how have the results improved equity? Have any of the reviews considered most relevant for equity in the process above been undertaken?
- Has there been an increase in authors from low- and middle-income settings?

Conclusion

A systematic and transparent approach to identifying review questions, informed by credible evidence, and reaching out to a broader group of people to assess priorities will help CEV establish which reviews need to be prioritised in the next 3 to 5 years.

For peer review only - http://bmjopen.bmj.com/site/about/guidelines.xhtml

For peer review only

References

1. Evans J, Li T, Virgili G, Wormald R, Cochrane E, Vision: Cochrane Eyes and Vision: a perspective introducing Cochrane Corner in Eye. *Eye* 2019, 33(6):882-886.

2. NICE: Age-related macular degeneration: diagnosis and management In: *NICE Guideline NG82.* National Institute of Health and Care Excellence; 2018.

3. NICE: Cataracts in adults: management. In: *NICE Guideline NG77.* National Institute of Health and Care Excellence; 2017.

4. Rowe F, Wormald R, Cable R, Acton M, Bonstein K, Bowen M, Bronze C, Bunce C, Conroy D, Cowan K *et al*: The Sight Loss and Vision Priority Setting Partnership (SLV-PSP): overview and results of the research prioritisation survey process. *BMJ open* 2014, 4(7):e004905.

5. Evans J, Mwangi N, Burn H, Ramke J: Equity was rarely considered in Cochrane Eyes and Vision systematic reviews and primary studies on cataract. *Journal of clinical epidemiology* 2020, 125:57-63.

6. Cochrane: Guidance note for Cochrane Groups to define systematic review priorities. In.; 2019.

7. Tong A, Synnot A, Crowe S, Hill S, Matus A, Scholes-Robertson N, Oliver S, Cowan K, Nasser M, Bhaumik S *et al*: Reporting guideline for priority setting of health research (REPRISE). *BMC medical research methodology* 2019, 19(1):243.

8. Burton MJ, Ramke J, Marques AP, Bourne RRA, Congdon N, Jones I, Ah Tong BAM, Arunga S, Bachani D, Bascaran C *et al*: The Lancet Global Health Commission on Global Eye Health: vision beyond 2020. *The Lancet Global health* 2021, 9(4):e489-e551.

9. WHO: World report on vision. . In. Geneva: World Health Organization

2019.

10. Keel S EJ, Block S, Bourne R, Calonge M, Cheng C-Y, Friedman DS, FurtadoJM, Khanna RC, Mathenge W, Mariotti S, Matoto E, Müller A, Rabiu MM, Rasengane T, Zhao J, Wormald R, Cieza A: Strengthening the integration of eye care into the health system: methodology for the development of the WHO package of eye care interventions. *BMJ Open Ophthalmology* 2020, 5(1).

11. Le J QR, Rouse B, Twose C, Rosman L, Lindsley K, Hawkins BS, Li T. : Development and content of a database of systematic reviews for eyes and vision. . *Eye* 2021, in press.

Acknowledgements

Cochrane Eyes and Vision Steering committee

- Michael Bowen (College of Optometrists)

For peer review only - http://bmjopen.bmj.com/site/about/guidelines.xhtml

- Roxanne Crosby-Nwaobi (Moorfields Eye Hospital)
- Jennifer Evans (Co-ordinating Editor, UK editorial base)
- Barny Foot (Royal College of Ophthalmologists)
- Stephen Gichuhi (University of Nairobi)
- Renata Gomes (Blind Veterans)
- John Lawrenson (City University)
- Tianjing Li (Co-ordinating Editor, CEV@US)
- Roses Parker (Cochrane Network Support Fellow)
- Jacqui Ramke (Editor with special interest in Equity)
- Fiona Rowe (Liverpool University)
- Anupa Shah (Managing Editor, UK editorial base)
- Gianni Virgili (Co-ordinating Editor, DTA satellite)
- Richard Wormald (Co-ordinating Editor, UK editorial base)

Author statement

All authors contributed to the conception and design of the study through participation in the Steering Group. JE wrote the first draft of the paper. JL, RP, FR GV, TL, JR revised it critically for important intellectual content. IG prepared the search strategies. All authors approved the submitted paper. As this is a protocol, acquisition, analysis and interpretation of data do not apply.

Funding statement

Cochrane Eyes and Vision is supported by the National Institute for Health Research (NIHR) Cochrane Infrastructure grant NIHR129461 until end March 2021 and Northern Ireland Health and Social Care Agency from April 1st 2021. Jacqueline Ramke's appointment at the University of Auckland is funded by the Buchanan Charitable Foundation, New Zealand.

Conflicts of interest

Tianjing Li directs the Cochrane Eyes and Vision US Project, supported by grant UG1EY020522 from the National Eye Institute, National Institutes of Health.

Word Count

2517

For peer review only - http://bmjopen.bmj.com/site/about/guidelines.xhtml

Figure 1: Flow-chart of study process

For peer review only

For peer review only - http://bmjopen.bmj.com/site/about/guidelines.xhtml

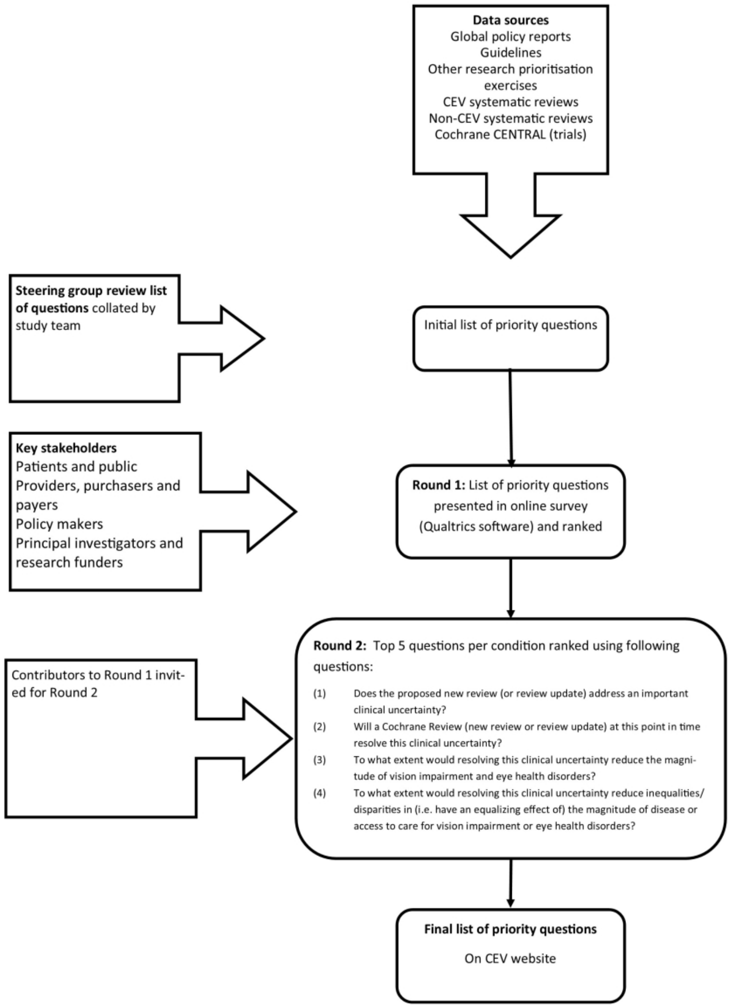

Figure 1: Flow-chart of study process

209x297mm (300 x 300 DPI)

Appendix 1: Search strategies

Roadmaps and other priority setting exercises (Google)

- roadmap ophthalmology
- unmet need ophthalmology
- priority setting ophthalmology
- prioritization research ophthalmology
- roadmap eye
- unmet need eye
- priority setting eye
- prioritization research eye

Cochrane Central register of controlled trials (CENTRAL)

- exp cataract and exp cataract extraction
- exp amblyopia/
- Strabismus/
- Ocular Motility Disorders/
- exp Nystagmus, Pathologic
- exp diabetic retinopathy
- exp eyelid diseases/
- Lacrimal Apparatus Diseases/
- Lacrimal Duct Obstruction/
- Nasolacrimal Duct/
- exp eye neoplasms
- exp glaucoma
- exp conjunctivitis/
- exp keratitis/
- exp uveitis/
- exp endophthalmitis/
- exp dry eye syndromes
- exp refractive errors
- vision, low
- visually impaired persons"
- blindness
- exp macular degeneration

For peer review only - http://bmjopen.bmj.com/site/about/guidelines.xhtml

Appendix 2: Information for participants / online survey

Cochrane Eyes and Vision Priority Setting Exercise

You are being invited to take part in a research study. Before you decide to take part please read the following information. If you are happy to participate, please tick the box below. Please contact Cochrane Eyes and Vision <email> if there is anything that is not clear or if you would like more information.

Purpose of the study: Cochrane Eyes and Vision plans to undertake a priority setting exercise to assess systematically the nature and extent of research questions relevant to our scope, and to formally incorporate input from a wider range of stakeholders to set priorities for new and updated reviews.

What is involved in taking part in the study? We would like you to take part in two rounds by online questionnaire. On the following pages, you will be presented with a list of potential review topics and asked to rank them in order of importance. These potential review topics were identified by systematic searching of global policy reports, guidelines and reports of relevant reviews and studies. We may have missed important questions and so there will also be an opportunity to tell us of priority topics that are not on the list.

There are separate surveys available for each eye condition (refractive error, cataract, glaucoma, eye problems in children, retinal conditions, outer eye, visual rehabilitation). Please select the condition(s) of interest. This is the first round and we expect it will take around 10 minutes per condition to do so.

Participants who provide their email address will be invited to take part in the second round. In the second round, you will be asked to rank the identified top priority topics according to four criteria. Based on responses to this survey, a final list with the highest ranked top 3 priority topics for each condition will be created.

Confidentiality: All information collected during the course of the research will be kept strictly confidential and will not be shared with anyone outside the research team. This information will be destroyed at the conclusion of the project. Your identity may be known to the researchers if you supply an identifiable email. All responses will be de-identified prior to analysis, and results will be reported at an aggregate level, so that your responses will not be identifiable as belonging to you.

What are the benefits? Participating in this study provides the opportunity to identify priorities for Cochrane Eyes and Vision reviews. These priorities may help guide eye health research and funding in future. Cochrane Eyes and Vision will prioritise the conduct of reviews on topics identified in this process. A summary of this priority setting exercise and the final list of priority topics will be published on Cochrane Eyes and Vision website and through relevant Cochrane channels, as well as in a scientific journal. If you provide your email address, we will also send you this information by email.

What are the risks? There are no risks of physical or psychological harm associated with this process. This first round is anticipated to take 10 minutes of your time at this stage, for each eye condition, and then the subsequent round will take approximately 15 minutes. You will not receive financial or

For peer review only - http://bmjopen.bmj.com/site/about/guidelines.xhtml

other type of reimbursement for taking part in the study. Participants who take part in both rounds will be acknowledged as part of group authorship, if they wish.

There is no obligation to take part, and if you do agree to take part you are still free to withdraw at any time and without giving a reason. This study has gained ethical approval from the London School of Hygiene and Tropical Medicine Research Ethics Committee.

Consent: I have read the information above and by ticking this box I consent to be involved in this study. I understand that at any time I may withdraw from this study without give a reason.

For peer review only

For peer review only - http://bmjopen.bmj.com/site/about/guidelines.xhtml
